# Treatment with Antihistamines and the Risk of Liver Cancer in Patients with Viral Hepatitis: A Multi-Center Cohort Study

**DOI:** 10.3390/v16060940

**Published:** 2024-06-11

**Authors:** Shu-Yen Chan, Yushan Chang, Natchaya Polpichai, Yuan-Ti Lee, Kevin Sheng-Kai Ma

**Affiliations:** 1 Department of Internal Medicine, Weiss Memorial Hospital/University of Illinois, Chicago, IL 60640, USA; chan0514.eras@gmail.com (S.-Y.C.); natchayap.md@gmail.com (N.P.); 2 Department of Medicine, National Cheng Kung University, Tainan 701401, Taiwan; yugi0604@gmail.com; 3 Division of Infectious Diseases, Department of Internal Medicine, Chung Shan Medical University Hospital, Taichung 402306, Taiwan; 4 School of Medicine, Chung Shan Medical University, Taichung 402306, Taiwan; 5 Center for Global Health, Perelman School of Medicine, University of Pennsylvania, Philadelphia, PA 19104, USA

**Keywords:** viral hepatitis, antihistamine, liver cancer, hepatitis C

## Abstract

**Background:** The effects of antihistamines on cancer risk and prognosis have been inconsistent across cancers. The aim of this multi-center cohort study was to investigate the association between antihistamine use and the risk of liver cancer in individuals with viral hepatitis. **Methods:** This multi-center cohort study included individuals diagnosed with hepatitis B or hepatitis C between January 2008 and March 2022. For antihistamine-treated patients, the index date was the date of antihistamine prescription, and for non-users, it was the date of hepatitis diagnosis. Participants were followed for five years, with the primary outcome of interest being new-onset liver cancer. The incidence rate and the adjusted hazard ratio (aHR) along with its 95% confidence interval (CI) of the outcome were calculated. Subgroup analyses were conducted, stratified by types of viral hepatitis including hepatitis C and hepatitis B. An additional validation study was performed. **Results:** The study included a total of 7748 patients with viral hepatitis. The incidence rate was 12.58 per 1000 person-years in patients with viral hepatitis on antihistamines, compared to 3.88 per 1000 person-years in those without antihistamine use. After adjusting for factors including age, sex, body mass index (BMI), comorbidities, laboratory data of liver function tests, comedications, and the use of antiviral therapies, the risk of new-onset liver cancer was significantly higher in patients on antihistamines (aHR = 1.83, 95% CI, 1.28–2.60). In patients with hepatitis C, the incidence rate in the antihistamine group was 15.73 per 1000 person-years, while non-users had a rate of 4.79 per 1000 person-years. Patients with hepatitis C on antihistamines had a significantly higher risk of developing liver cancer (aHR = 3.24, 95% CI, 2.16–4.86). **Conclusions:** This multi-center cohort study reported an increased risk of liver cancer in patients with hepatitis B or hepatitis C treated with antihistamines. Long-term follow-up studies are warranted to validate the findings.

## 1. Introduction

Hepatitis is characterized by inflammation of the liver and can arise from diverse factors including excessive alcohol consumption, autoimmune conditions, drug reactions, or exposure to toxins. Nevertheless, the primary cause of hepatitis is commonly attributed to viral infections [1]. According to a global report, the prevalence of hepatitis B virus (HBV) was reported to be approximately 3.9% in 2016 [2]. As for hepatitis C, the viremic prevalence was reported to be 0.78% in women and 0.13% in children [3,4]. An association between viral hepatitis and subsequent liver cancer incidence has been well developed. Chronic viral hepatitis can result in severe liver damage, encompassing conditions such as liver fibrosis, cirrhosis, and hepatocellular carcinoma. These outcomes contribute to substantial morbidity and mortality associated with the chronic form of the disease [5,6]. Given the profound impact of liver cancer on individuals’ quality of life, it is crucial to address interventions for people with viral hepatitis and examine their potential impact on the subsequent development of liver cancer.

Serving as a critical substance regulating and enhancing inflammatory responses, the role of histamine in the development of immune responses has long been discussed [7]. Immunological reactions are mediated based on different antagonists on histamine receptors. Given that malignancies are massively related to long-term inflammation status, related anti-inflammatory treatments such antihistamines have been considered a potential option for therapies for malignancies [8,9]. In previous studies, antihistamine use has been reported to have potential anti-neoplasm effects [10,11,12]. That said, the influences of antihistamines on the development of liver cancer have not been well investigated. While a cohort study reported that antihistamine utilization was associated with a decreased risk of future hepatocellular carcinoma in people with hepatitis B and C [13], its clinical evidence is limited due to a lack of information regarding, laboratory data, indication of diseases for antihistamine use, and body mass index (BMI) that should be considered in the analyses.

The utilization of antihistamines and the advancement of chronic hepatitis-related conditions, such as liver cirrhosis and liver cancer, may both contribute to the manifestation of pruritus symptoms. Consequently, physicians may contemplate prescribing antihistamines as part of the treatment regimen. During the course of treatment for hepatitis B and hepatitis C, patients undergoing interferon therapy or treatment with direct antiviral agents (DAAs) may experience pruritus symptoms as well, thus prompting physicians to consider prescribing antihistamines. Given the common prescriptions of antihistamines in patients with viral hepatitis, the aim of this multi-center cohort study was to identify the association between antihistamine use and the risk of liver cancer in patients with viral hepatitis.

## 2. Materials and Methods

Electronic health records from three branches of the Chung Shan Medical University Hospital (Taichung, Taiwan) were retrieved. The dataset has been used and described in previous studies [14,15,16]. In the main analysis, people ≥ 18 years old with a diagnosis of viral hepatitis between January 2008 and March 2022 were included as the study population. For antihistamine-treated patients, the index date was the date of antihistamine prescription, while for non-users, it was the date of hepatitis B or C diagnosis. Participants were followed for a maximum of five years, with the primary outcome of interest being new-onset liver cancer. Participants were excluded based on the following criteria: (1) having a previous history of any cancer, (2) being diagnosed with liver cancer after less than 1 year of the hepatitis diagnosis, and (3) having a previous record of hepatectomy. Patients with missing data were censored.

Incidences of the outcome event were calculated in both antihistamine users and non-users. The Kaplan-Meier curves were plotted to demonstrate the effect of exposure on outcomes over time. A *p*-value < 0.05 represents a statistically significant difference between two groups. The crude hazard ratio (cHR) and adjusted hazard ratio (aHR) and 95% confidence interval (95% CI) were calculated in the multivariable Cox regression model to evaluate the effect of antihistamines on the risk of outcome over time. Covariates adjusted in the multivariate Cox model included age, sex, BMI, lab data related to liver function (including aspartate aminotransferase [AST], alanine aminotransferase [ALT], gamma-glutamyl transpeptidase [r-GT], globulin, albumin to globulin [A/G] ratio, and total protein), previous treatments such as liver transplantation and radiotherapy, comorbidities (allergic rhinitis, asthma, pruritis, prurigo, hepatitis B, hepatitis C, cirrhosis, alcoholic liver disease, diabetes mellitus, hypertension, chronic kidney diseases, and non-alcoholic fatty liver disease), and comedications (non-aspirin non-steroidal anti-inflammatory drugs, aspirin, statin, antineoplastic agents including cisplatin, fluorouracil, doxorubicin, and antiviral agents including adefovir, lamivudine, telbivudine, entecavir, peginterferon, and ribavirin). Two additional cohorts based on the following models were constructed for sensitivity analysis to evaluate the detailed association between antihistamine use and liver cancer development: (1) Participants were limited to those having only hepatitis C. Patients with hepatitis B during the observation period were excluded from the analysis. (2) Participants were limited to those having only hepatitis B. Patients with hepatitis C during the observation period were excluded from the analysis. In the main analysis and sensitivity analyses, study designs and adjusted covariates were based on identical settings.

To validate the effect of antihistamines among patients with viral hepatitis, an additional study was conducted using electronic health records from a collaborative network in the United States [17,18] (TriNetX, LLC, Cambridge, MA, USA). In the validation study, the risk of outcomes was compared between the exposed group and the non-exposed group. The primary outcomes were all-cause mortality, liver cancer, ascites, cirrhosis, and hepatic encephalopathy. Covariates such as demographics, comorbidities, antiviral agents, and comedications were propensity score matched between the antihistamine and non-antihistamine groups. The hazard ratio (HR) along with its 95% CI and the *p*-value of log-rank tests were calculated for each outcome. Methods of the validation study has been described in previous studies [19,20,21,22].

## 3. Results

A total of 7748 patients with hepatitis B or C were included, with 3658 viral hepatitis patients in the antihistamine group and 4090 viral hepatitis patients in the non-antihistamine group (Table 1). The mean age of people in the antihistamine group was 56 years old, whereas in the non-antihistamine group, the mean age was 51. The male percentage was higher in the antihistamine group than in the control group, at 44.2% and 40.4%, respectively. For antihistamine users, the mean BMI was 24, and for non-users, the mean BMI was 25. Laboratory data measured at baseline, including AST, r-GT, globulin, and A/G ratio, were similar between the two groups.

**Figure 1:**
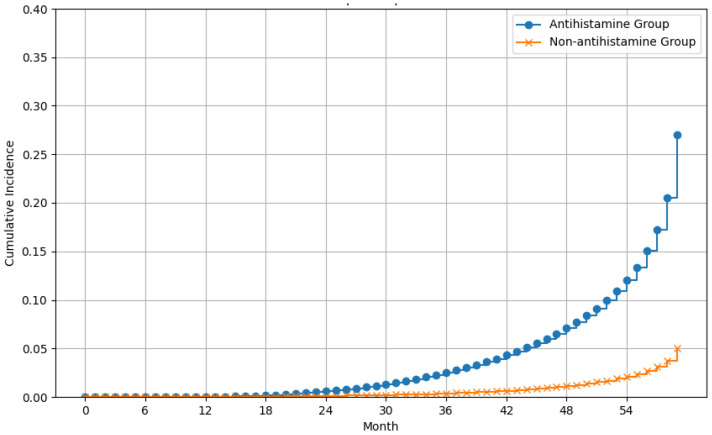
Effect of antihistamines on liver cancer risk in patients with hepatitis C or hepatitis B over time.

The incidence rates of liver cancer were 12.58 per 1000 person-years in viral hepatitis patients using antihistamines and 3.88 per 1000 person-years in those without antihistamine use. The risk of liver cancer was significantly higher in antihistamine users with viral hepatitis than did non-users (cHR = 3.21, 95% CI, 2.33–4.41). The finding was consistent after adjusting for age, sex, BMI, comorbidities, laboratory tests, comedications, and related therapies (aHR = 1.83, 95% CI, 1.28–2.60) (Table 2; Figure 1). The use of antiviral agents did not significantly influence the risk of new-onset liver cancer (Table 2).

**Figure 2:**
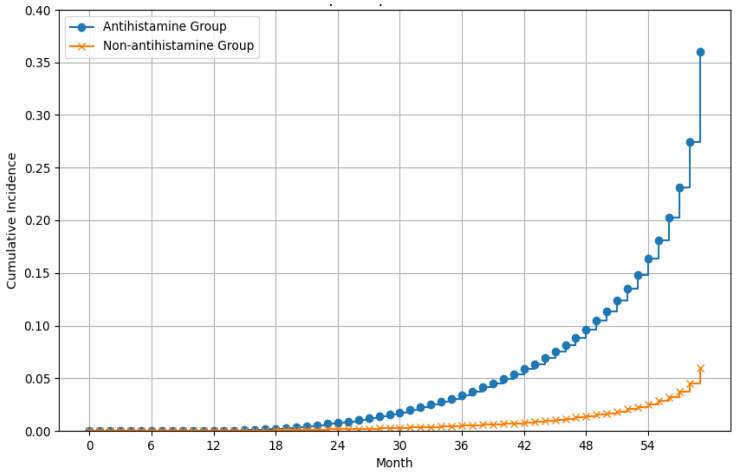
Effect of antihistamines on liver cancer risk in patients who only had hepatitis C over time.

Among patients who only had hepatitis C, there were 2055 antihistamine users and 2158 non-users. The incidence rate in the antihistamine group was 15.73 per 1000 person-years. Compared to hepatitis C patients never exposed to antihistamines, those receiving antihistamines presented with a significantly higher risk of liver cancer (cHR = 3.24, 95% CI, 2.16–4.86; aHR = 2.23, 95% CI, 1.43–3.48) (Table 3) (Figure 2).

**Figure 3:**
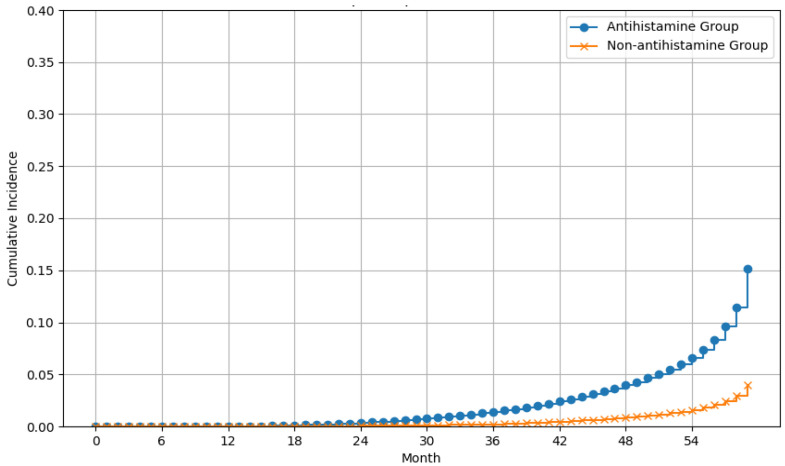
Effect of antihistamines on liver cancer risk in patients who only had hepatitis B over time.

Among patients who only had hepatitis B (Table 3), the incidence rates of liver cancer were 6.37 for antihistamine users and 2.73 for non-users. After adjusting for covariates, the risk of liver cancer did not reach statistical significance in patients with hepatitis B who received antihistamines (aHR = 1.22, 95% CI, 0.61–2.47) (Figure 3).

In the validation study, baseline covariates, including demographics, comorbidities, and comedications such as antiviral agents (including ribavirin, sofosbuvir, peginterferon, ledipasvir, glecaprevir, pibrentasvir, dasabuvir, entecavir, tenofovir disoproxil), were balanced in two groups (Supplementary Tables S1 and S2). Among patients who only had HCV infections, antihistamine users were consistently associated with a significantly higher risk of liver cancer (HR: 2.23, 95% CI, 1.58–3.16) (Table 4). Additionally, antihistamine users with HCV infections were also found to have a significantly increased risk of hepatic encephalopathy (HR: 1.92, 95% CI, 1.14–3.23) and ascites (HR: 1.29, 95% CI, 1.08–1.54). Among patients who only had HBV infections, no significant differences in all-cause mortality and liver cancer risk were found between antihistamine users and non-user groups (Table 5).

## 4. Discussion

This multi-center cohort study revealed an elevated risk of liver cancer in individuals with hepatitis B or hepatitis C treated with antihistamines. The risk trend surpassed a twofold increase when focusing solely on people with hepatitis C. Findings from this observational study raise concerns about using antihistamines in patients with both viral hepatitis and liver cancer, emphasizing the need for further research to elucidate the underlying mechanisms.

The possible mechanism could be related to certain inflammatory responses resulting from chronic hepatitis, leading physicians to prescribe antihistamines. Or it is also possible that the use of antihistamines may cause serious complications. Both possibilities require further research for confirmation. Histamine plays various roles in cancer progression. In various cancers, including melanoma and non-small cell lung cancer, the activity of histidine decarboxylase, an enzyme that is highly involved in histamine secretion, has been reported to be significantly increased in laboratory studies [23,24,25]. While histamine involved in several mechanisms regarding inflammation and malignancy progression, real-world studies do not always support the notion that antihistamines decrease the risk of cancer development. A Danish case–control study stated that antihistamine use did not influence the risk of developing ovarian cancer, despite the effect of antihistamines on lowering ovarian cancer risk being observed in pre-menopause women. Likewise, a cohort study reported an insignificant association between antihistamine use and contralateral breast cancer risk [26]. As for the involvement of antihistamine and hepatocellular cancerous cells, previous studies based on cell and animal models reported blocked autophagy pathways in hepatoma cell death [27]. Although liver diseases were thought to be associated with long-term inflammatory status [28,29], the relation between antihistamine use and liver cancer development were not clearly discussed in previous studies, and only few observational results based were available [13].

In the current study, an increased risk of liver cancer was observed in viral hepatitis patients using antihistamines. The association could potentially be attributed to the previously reported cancer-inducing mechanism of antihistamines and the potential immune-stimulatory function of H1 antihistamines. In a rodent model, tumor growth factor could be triggered due to the structural similarity between antihistamines and DPPE [*N*,*N*-diethyl-2-(4-(phenylmethyl)phenoxy) ethanamine HC1] [30,31]. Due to the interaction with the immune system, the functions of histamine were not limited to merely immune-stimulatory but also immune-suppressive functions on different occasions [32]. H1 receptors could function as modulators of NF-κB pathway. Therefore, through activating H1 receptors, inflammatory-related substances such as leukotrienes and prostaglandins could be secreted and lead to inflammatory reactions [30]. It was stated that although antihistamines were able to provide anti-inflammatory functions, inflammatory reactions could still be triggered since H1 receptor has been aroused [31]. Given that H1 receptors and histamine itself could potentially lead to the stimulation of inflammation pathways, H1 antihistamines were also reported to be associated with an increased risk of other cancers [31].

In the present study, the indications for antihistamines were adjusted as covariates in the multivariate Cox regression analysis. Some of these comorbidities were associated with endocrinological or cancerous events, which could serve as potential confounders in the evaluation of the outcome event [33,34]. Comedications including interferon and DAAs were also considered confounders due to their potential influence on liver cancer incidence [35,36]. The increasing risk of liver cancer in antihistamine users was especially observed in people with hepatitis C. In the subgroup of patients who only had hepatitis B, although there was statistical significance in the crude models, the statistical significance did not remain after adjusting potential confounders. Neither result supports the hypothesis that antihistamine use could serve as protective factor against liver cancer incidence, which was inconsistent with two previous studies [13,37]. The results of the present study were further validated by observations based on an external data source. Some of the detailed data such as BMI and AST, ALT, and albumin information was not accessible in previous real-world studies; moreover, potential confounders including hepatectomy records and anti-neoplasm agent use were also unconsidered. We suppose the trend of liver cancer incidence, which was different from that in previous reports [12], could be partially attributed to the potential effect of confounding by indication and residual confounders. Additionally, a recent population-based cohort study also suggested a significant association between antihistamine use and an increased risk of esophageal squamous cell carcinoma, with a dose–response relationship [38].

The strengths of the present study included a large cohort of patients from multiple hospitals and the adjustment of confounders, including age, sex, comorbidities, laboratory data of liver function tests, and comedications. To mitigate potential confounding by indication, diseases with antihistamine indications were incorporated as covariates [39,40]. Furthermore, sensitivity and validation tests were conducted, which yielded consistent results. That said, several limitations should be acknowledged. These include the inability to measure over-the-counter comedication use and to calculate the cumulative defined daily dosage and duration of antihistamine usage accurately. Moreover, the lack of data on viral load and immune status within the datasets precluded their inclusion in the analyses, limiting insights into their potential influence on observed trends in liver cancer risk. Furthermore, residual confounders may exist despite adjustment of covariates.

## 5. Conclusions

This study found an increased risk of liver cancer in people with viral hepatitis using antihistamines, especially for those with hepatitis C. Future randomized controlled trials or large-scale, population-based studies are needed to validate the association between antihistamine and future malignancy development.

## Figures and Tables

**Table 1:** Baseline characteristics of patients with viral hepatitis on antihistamine users and antihistamine non-users.

Characteristics	Antihistamine Users with Viral Hepatitis		Non-Users with Viral Hepatitis		*p*-Value
	(*n* = 3658)		(*n* = 4090)		
	*n*	%	*n*	%	
Age at index date	56 ± 15	-	51 ± 14	-	<0.001
Female	2040	55.77	2439	59.63	<0.001
Male	1618	44.23	1651	40.37	<0.001
BMI ^1^ (Mean ± SD)	24 ± 4	-	25 ± 4	-	<0.01
Comorbidities					
Allergic rhinitis	798	21.82	170	4.16	<0.001
Asthma	248	6.78	42	1.03	<0.001
Pruritis	193	5.28	32	0.78	<0.001
Prurigo	192	5.25	28	0.68	<0.001
HBV infection	1528	41.77	1838	44.94	<0.01
HCV infection	1143	31.25	794	19.41	<0.001
Cirrhosis	1073	29.33	514	12.57	<0.001
Alcoholic liver disease	296	8.09	114	2.79	<0.001
Diabetes mellitus	1144	31.27	646	15.79	<0.001
Hypertension	1694	46.31	940	22.98	<0.001
Chronic kidney diseases	784	21.43	241	5.89	<0.001
NAFLD	422	11.54	313	7.65	<0.001
Antiviral agents					
Adefovir	30	0.82	19	0.46	<0.05
Lamivudine	214	5.85	125	3.06	<0.001
Telbivudine	26	0.71	22	0.54	0.33
Entecavir	399	10.91	269	6.58	<0.001
Peginterferon	441	12.06	183	4.47	<0.001
Ribavirin	484	13.23	194	4.74	<0.001
Other comedications					
NSAIDs	2452	67.03	1478	36.14	<0.001
Statin	947	25.89	500	12.22	<0.001
Aspirin	396	10.83	138	3.37	<0.001
Cisplatin	28	0.77	3	0.07	<0.001
Fluorouracil	33	0.90	3	0.07	<0.001
Doxorubicin	6	0.16	0	0.00	<0.01
Liver function-related procedures					
Liver transplantation	15	0.41	2	0.05	<0.001
Radiotherapy	47	1.28	3	0.07	<0.001
Lab data					
AST (mean ± SD)	141 ± 356	-	133 ± 227	-	0.43
ALT (mean ± SD)	124 ± 297	-	152 ± 305	-	<0.01
r-GT (mean ± SD)	203 ± 234	-	209 ± 250	-	0.64
Globulin (mean ± SD)	4 ± 1	-	4 ± 0	-	0.07
Total protein (mean ± SD)	7 ± 2	-	7 ± 1	-	<0.05
A/G ratio (mean ± SD)	1 ± 0	-	1 ± 0	-	0.71

^1^ BMI, body mass index; HBV, hepatitis B virus; HCV, hepatitis C virus; NAFLD, non-alcoholic fatty liver disease; AST, aspartate aminotransferase; ALT, alanine aminotransferase; r-GT, G gamma-glutamyl transpeptidase; A/G ratio, albumin to globulin ratio; SD, standard deviation.

**Table 2:** Risk of liver cancer in patients with hepatitis B or C.

Variable		Crude HR ^1^ (95%CI)	aHR ^2^ (95%CI)
Antihistamine use			
	No	Reference	Reference
	Yes	3.21 (2.33–4.41)	1.83 (1.28–2.60)
Sex			
	Female	Reference	Reference
	Male	0.84 (0.65–1.07)	1.14 (0.85–1.51)
Age	Increased risk per unit	1.06 (1.05–1.07)	1.04 (1.03–1.06)
BMI	Increased risk per unit	0.99 (0.92–1.05)	0.99 (0.97–1.00)
Comorbidities			
	Allergic rhinitis	0.61 (0.42–0.89)	0.75 (0.50–1.12)
	Asthma	0.83 (0.45–1.51)	0.58 (0.31–1.08)
	Pruritis	1.68 (1.03–2.75)	1.19 (0.70–2.01)
	Prurigo	0.96 (0.51–1.81)	0.82 (0.42–1.58)
	Cirrhosis	13.59 (10.00–18.45)	7.80 (5.59–10.88)
	Alcoholic liver diseases	3.45 (2.40–4.96)	1.67 (1.08–2.58)
	Diabetes mellitus	2.17 (1.69–2.79)	1.36 (1.03–1.79)
	Hypertension	1.82 (1.41–2.34)	0.88 (0.66–1.17)
	Chronic kidney disease	1.72 (1.28–2.30)	0.86 (0.62–1.19)
	Non-alcoholic fatty liver diseases	0.96 (0.65–1.41)	0.95 (0.64–1.41)
Antiviral agents			
	Adefovir	0.99 (0.32–3.09)	1.01 (0.28–3.68)
	Lamivudine	0.78 (0.41–1.47)	1.06 (0.51–2.21)
	Telbivudine	1.19 (0.38–3.70)	1.36 (0.42–4.39)
	Entecavir	1.14 (0.78–1.66)	1.08 (0.69–1.68)
	Peginterferon	1.00 (0.70–1.43)	0.86 (0.46–1.60)
	Ribavirin	1.34 (0.96–1.82)	0.88 (0.50–1.54)
Antineoplastic agents			
	Cisplatin	2.43 (0.90–6.52)	0.24 (0.07–0.89)
	Fluorouracil	4.47 (2.21–9.04)	5.06 (2.12–12.12)
	Doxorubicin	3.07 (0.43–21.87)	0.69 (0.09–5.34)
Other comedications			
	NSAIDs	1.10 (0.85–1.44)	0.99 (0.75–1.31)
	Statin	0.52 (0.37–0.74)	0.49 (0.34–0.72)
	Aspirin	1.46 (1.00–2.13)	0.89 (0.59–1.33)
Lab tests			
	Globulin	1.78 (1.11–2.87)	1.78 (1.11–2.87)
	A/G ratio	0.23 (0.05–1.11)	0.23 (0.05–1.11)
	Total protein	1.02 (0.81–1.28)	1.02 (0.81–1.28)
Other therapy			
	Radiotherapy	5.82 (3.33–10.18)	4.25 (2.15–8.41)
	Liver transplantation	7.93 (3.27–19.22)	2.07 (0.83–5.16)

^1^ HR, hazard ratio; aHR, adjusted hazard ratio; BMI, body mass index; HBV, hepatitis B virus; HCV, hepatitis C virus; NSAIDs, non-steroidal anti-inflammatory drugs; globulin (≥3.5 g/dL); A/G ratio, albumin/globulin ratio (≤1); total protein (≥8 g/dL or ≤6 g/L). ^2^ Adjusted age, sex, BMI, comorbidities, lab tests, comedications, and related therapies.

**Table 3:** Incidence and risk of liver cancer in patient with viral hepatitis, patients who only had hepatitis C, and patients who only had hepatitis B.

Antihistamine Users with Hepatitis B or C	Number of Patients	Number of Events	Person-Years	Incidence Rate ^1^	Crude HR	Adjusted HR ^2^
Antihistamine users	3658	198	15,738.51	12.58	3.21 (2.33–4.41)	1.83 (1.28–2.60)
Antihistamine non-users	4090	47	12,106.52	3.88	Reference	Reference
Antihistamine users who only hadhepatitis C	Number of Patients	Number of Events	Person-Years	Incidence Rate	Crude HR	Adjusted HR
Antihistamine users	2055	144	9152.96	15.73	3.24 (2.16–4.86)	2.23 (1.43–3.48)
Antihistamine non-users	2158	28	5840.23	4.79	Reference	Reference
Antihistamine users whoonly had hepatitis B	Number of Patients	Number of Events	Person-Years	Incidence Rate	Crude HR	Adjusted HR
Antihistamine users	1437	37	5808.69	6.37	2.32 (1.29–4.17)	1.22 (0.61–2.47)
Antihistamine non-users	1826	16	5863.19	2.73	Reference	Reference

^1^ Per 1000 person-years. ^2^ Adjusted age, sex, BMI, comorbidities, lab tests, comedications, and related therapies.

**Table 4:** Comparison of outcomes in patients who only had chronic HCV infections after propensity score matching.

	Antihistamine Users (*n* = 2855)	Non-Users (*n* = 2855)	HR	95% CI	*p*-Value (Log-Rank Test)
All-cause mortality	399	384	0.93	(0.81, 1.07)	0.29
Liver cancer	109	45	2.23	(1.58, 3.16)	0.00
Hepatic encephalopathy	44	21	1.92	(1.14, 3.23)	0.01
Ascites	300	215	1.29	(1.08, 1.54)	0.00
Cirrhosis	737	630	1.10	(0.99, 1.22)	0.09

HR, hazard ratio; CI, confidence interval.

**Table 5:** Comparison of outcomes in patients who only had chronic HBV infections after propensity score matching.

	Antihistamine Users (*n* = 317)	Non-Users (*n* = 317)	HR	95% CI	*p*-Value (Log-Rank Test)
All-cause mortality	25	29	0.75	(0.44, 1.29)	0.30
Liver cancer	10	10	4.64	(0.54, 39.73)	0.12
Hepatic encephalopathy	10	10	1.10	(0.43, 2.79)	0.84
Ascites	42	25	1.57	(0.96, 2.58)	0.07
Cirrhosis	72	68	0.96	(0.69, 1.34)	0.83

HR, hazard ratio; CI, confidence interval.

## Data Availability

The raw data supporting the conclusions of this article will be made available by the corresponding author, K.S.-K.M., on request. The data are not publicly available due to hospital policy and patients’ privacy.

## Supplementary Materials

The following supporting information can be downloaded at: https://www.mdpi.com/article/10.3390/v16060940/s1. Table S1. Baseline characteristics in patients with HCV chronic infection on antihistamines and those who did not use antihistamines. Table S2. Baseline characteristics in patients with HBV chronic infection on antihistamines and those who did not use antihistamines.
