# Supplementary material for: Treatment with Antihistamines and the Risk of Liver Cancer in Patients with Viral Hepatitis: A Multi-Center Cohort Study
Source: Viruses. 2024 Jun 11;16(6):940. doi: 10.3390/v16060940 (PMC11209559; doi:10.3390/v16060940)
Supplement: Supplementary file 1 [file viruses-16-00940-s001.zip › viruses-3040033-supplementary.pdf]

viruses MDPI

Supplementary Materials:

Table S1*. Baseline characteristics in patients with HCV chronic infection on antihistamines and those who did not use antihistamines*

	Before propensity score matching			After propensity score matching		
	With antihistamine (4125)	Without antihistamine (7860)	SMD	With antihistamine (2855)	Without antihistamine (2855)	SMD
**Age at Index**						
Mean ± SD	53.7 ± 12.2	50.6 ± 12.4	0.26	52.5 ± 12.4	52.5 ± 12.4	0.01
**Demographic, n (%)**						
Male	1972 (48.09)	4361 (57.17)	0.18	1419 (49.70)	1422 (49.81)	0.00
Female	2001 (48.79)	3017 (39.55)	0.19	1348 (47.22)	1353 (47.39)	0.00
Black or African American	1028 (25.07)	1619 (21.22)	0.09	672 (23.54)	645 (22.59)	0.02
White	1731 (42.21)	3825 (50.14)	0.16	1292 (45.25)	1313 (45.99)	0.01
Asian	200 (4.88)	244 (3.199)	0.09	136 (4.76)	129 (4.52)	0.01
Unknown Race	991 (24.17)	1580 (20.71)	0.08	646 (22.63)	658 (23.05)	0.01
Not Hispanic or Latino	1775 (43.28)	3038 (39.83)	0.07	1252 (43.85)	1248 (43.71)	0.00
Hispanic or Latino	306 (7.46)	609 (7.984)	0.02	226 (7.92)	232 (8.13)	0.01
**Comorbidities, n (%)**						
Asthma	918 (22.39)	762 (9.99)	0.34	488 (17.09)	491 (17.20)	0.00
Allergic rhinitis	578 (14.09)	360 (4.719)	0.33	284 (9.95)	263 (9.21)	0.03
Pruritus	482 (11.75)	234 (3.068)	0.34	190 (6.66)	178 (6.24)	0.02
Prurigo	47 (1.15)	43 (0.564)	0.06	20 (0.70)	21 (0.74)	0.00
Type 2 diabetes mellitus	1370 (33.41)	1407 (18.45)	0.35	780 (27.32)	781 (27.36)	0.00
Chronic kidney disease	844 (20.58)	567 (7.433)	0.39	426 (14.92)	406 (14.22)	0.02
Alcoholic liver disease	44 (1.07)	56 (0.734)	0.04	28 (0.98)	28 (0.98)	<0.0001
NAFLD	391 (9.53)	268 (3.513)	0.25	170 (5.95)	165 (5.78)	0.01
Essential hypertension	2516 (61.35)	2892 (37.91)	0.48	1534 (53.73)	1540 (53.94)	0.00
**Liver function-related procedures**						
Liver transplantation	15 (0.37)	10 (0.131)	0.05	10 (0.35)	10 (0.35)	< 0.0001
Radiotherapy	29 (0.71)	15 (0.197)	0.08	13 (0.46)	10 (0.35)	0.02
**Antiviral agents**						
Ribavirin	500 (12.19)	345 (4.523)	0.28	249 (8.72)	240 (8.41)	0.01
Sofosbuvir	468 (11.41)	145 (1.901)	0.39	158 (5.53)	135 (4.73)	0.04
Peginterferon	245 (5.97)	162 (2.124)	0.20	119 (4.17)	120 (4.20)	0.00
Ledipasvir	305 (7.44)	92 (1.206)	0.31	106 (3.71)	84 (2.94)	0.04
Glecaprevir	58 (1.41)	11 (0.144)	0.14	10 (0.35)	10 (0.35)	< 0.0001
Pibrentasvir	51 (1.24)	10 (0.131)	0.09	10 (0.35)	10 (0.35)	< 0.0001
Dasabuvir	41 (1)	11 (0.144)	0.11	12 (0.42)	11 (0.39)	0.01
Ombitasvir	39 (0.95)	11 (0.144)	0.11	12 (0.42)	11 (0.39)	0.01
Paritaprevir	39 (0.95)	11 (0.144)	0.11	12 (0.42)	11 (0.39)	0.01
Elbasvir	30 (0.73)	10 (0.131)	0.09	10 (0.35)	10 (0.35)	< 0.0001
**Other Medications**						
NSAIDs	2715 (66.20)	2516 (32.98)	0.70	1689 (59.16)	1722 (60.32)	0.02
Statin	1090 (26.58)	836(10.96)	0.41	578 (20.25)	591 (20.70)	0.01
Aspirin	1443 (35.19)	1117 (14.64)	0.49	777 (27.22)	764 (26.76)	0.01
Cisplatin	10 (0.24)	10 (0.131)	0.03	10 (0.35)	10 (0.35)	< 0.0001
Fluorouracil	10 (0.24)	10 (0.131)	0.03	10 (0.35)	10 (0.35)	< 0.0001
Doxorubicin	10 (0.24)	10 (0.131)	0.03	10 (0.35)	10 (0.35)	< 0.0001
**Laboratory, mean ± SD**						
BMI (kg/m2) [a]	28.6 ± 6.61	27.5 ± 5.88	0.17	28.2 ± 6.53	27.9 ± 6.06	0.05
AST (mean ± SD)	44.5 ± 57.1	60.2 ± 102	0.19	46.5 ± 55.5	51.8 ± 65.2	0.09
ALT (mean ± SD)	48.9 ± 75.2	57 ± 86.7	0.10	49.6 ± 54.9	52.3 ± 85.3	0.04
Total rotein (mean ± SD)	7.28 ± 0.87	7.31 ± 0.82	0.04	7.29 ± 0.85	7.29 ±0.84	0.04
r-GT (mean ± SD)	111 ± 182	109 ± 196	0.01	106 ± 166	110 ± 200	0.02
Globulin (mean ± SD)	3.42 ± 0.80	3.35 ± 0.76	0.09	3.42 ± 0.82	3.38 ± 0.73	0.04
A/G ratio (,ean ± SD)	1.17 ± 0.41	1.25 +/- 0.39	0.20	1.19 ± 0.45	1.22 ± 0.39	0.07
HCV RNA [Units/volume] (mean ± SD)	1088693 ± 2313074	1457086 ± 2720407	0.15	1231785 ± 2509168	1389738 ± 2835559	0.06

BMI, body mass index; NAFLD, non-alcoholic fatty liver disease; NSAIDs, non-steroidal anti-inflammatory drugs; AST, aspartate aminotransferase (U/L); ALT, alanine aminotransferase (U/L); total protein (g/L); r-GT, gamma-glutamyl transpeptidase (U/L); A/G ratio, albumin to globulin ratio; HCV RNA, hepatitis virus C Ribonucleic Acid (IU/mL); SD, standard deviation

Table S2. *Baseline characteristics in patients with HBV chronic infection on antihistamines and those who did not use antihistamines*

	Before propensity score matching			After propensity score matching		
	With antihistamine (377)	Without antihistamine (8050)	SMD	With antihistamine (317)	Without antihistamine (317)	SMD
**Age at Index**						
Mean ± SD	52.3 ± 13.3	47.2 ± 14.6	0.36	51.9 +/- 13.5	51.0 +/- 14.7	0.06
**Demographic, n (%)**						
Male	208 (55.2)	4,409 (57.0)	0.04	171 (53.9)	158 (49.8)	0.08
Female	164 (43.5)	3,233 (41.8)	0.03	141 (44.5)	155 (48.9)	0.09
Black or African American	123 (32.6)	1,935 (25.0)	0.17	97 (30.6)	107 (33.8)	0.07
White	93 (24.7)	1801 (23.3)	0.03	77 (24.30)	70 (22.1)	0.05
Asian	100 (26.5)	2406 (31.1)	0.10	89 (28.1)	93 (29.3)	0.03
Unknown race	38 (10.1)	1110 (14.4)	0.13	32 (10.1)	24 (7.6)	0.09
Not Hispanic or Latino	340 (90.2)	6399 (82.8)	0.22	284 (89.6)	298 (94.0)	0.16
Hispanic or Latino	21 (5.6)	480 (6.2)	0.02	18 (5.7)	10 (3.2)	0.12
**Comorbidities, n (%)**						
Asthma	54 (14.3)	352 (4.6)	0.34	43 (13.6)	47 (14.8)	0.04
Allergic rhinitis	48 (12.7)	196 (2.5)	0.39	34 (10.7)	41 (12.9)	0.07
Pruritus	58 (15.4)	251 (3.2)	0.43	44 (13.9)	41 (12.9)	0.03
Prurigo	10 (2.7)	24 (0.3)	0.20	10 (3.2)	10 (3.2)	<0.0001
Type 2 diabetes mellitus	143 (37.9)	1067 (13.8)	0.57	108 (34.1)	111 (35.0)	0.02
Chronic kidney disease	180 (47.7)	650 (8.4)	0.97	131 (41.3)	126 (39.7)	0.03
Alcoholic liver disease	10 (2.7)	21 (0.3)	0.20	10 (3.2)	10 (3.2)	< 0.0001
NAFLD	72 (19.1)	444 (5.7)	0.41	61 (19.2)	57 (18.0)	0.03
Essential hypertension	255 (67.6)	1919 (24.8)	0.95	201 (63.4)	197 (62.1)	0.03
**Liver function-related procedures**						
Liver transplantation	27 (7.24)	11 (0.15)	0.38	12 (3.8)	10 (3.2)	0.03
Radiotherapy	10 (2.68)	10 (0.13)	0.22	10 (3.2)	10 (3.2)	<0.0001
**Antiviral agents**						
adefovir	10 (2.7)	23 (0.30)	0.20	10 (3.2)	10 (3.2)	<0.0001
lamivudine	27 (7.2)	107 (1.4)	0.29	20 (6.3)	15 (6.0)	0.01
telbivudine	10 (2.7)	10 (0.1)	0.22	0 (0)	10 (3.2)	0.26
entecavir	113 (30.30)	296 (3.8)	0.74	76 (24.0)	70 (22.1)	0.05
Tenofovir disoproxil	119 (31.6)	561 (7.3)	0.65	84 (26.5)	90 (28.4)	0.04
**Other Medications**						
NSAIDs	208 (55.2)	1597 (20.7)	0.76	171 (53.9)	176 (55.5)	0.03
Statin	134 (35.5)	944 (12.2)	0.57	109 (34.4)	121 (38.2)	0.01
Aspirin	170 (45.1)	691 (8.9)	0.89	125 (39.4)	115 (36.3)	0.07
Cisplatin	10 (2.7)	0 (0)	0.23	0 (0)	0 (0)	-
Fluorouracil	10 (2.7)	10 (0.1)	0.22	10 (3.2)	10 (3.2)	<0.0001
Doxorubicin	10 (2.7)	0 (0)	0.23	10 (3.2)	0 (0)	0.26
**Laboratory, Mean ± SD**						
BMI (kg/m2) [a]	26.9 ± 6.3	26.8 ± 6.1	0.02	27.0 ± 6.3	27.8 ± 6.3	0.12
AST (mean ± SD)	58.0 ± 158.5	63.9 ± 249.9	0.03	54.6 ± 161.1	62.2 ± 154.9	0.05
ALT (mean ± SD)	63.5 ± 179.8	56.4 ± 193.6	0.04	60.4 ± 187.5	53.7 ± 107.6	0.04
Total rotein (mean ± SD)	7.0 ± 1.0	7.3 ± 0.8	0.31	7.0 ± 1.0	7.2 ± 0.9	0.20
r-GT (mean ± SD)	109.8 ± 176.8	87.2 ± 208.1	0.12	83.9 ± 151.8	106.5 ± 191.0	0.13
Globulin (mean ± SD)	3.3 ± 0.9	3.2 ± 0.7	0.19	3.3 ± 0.9	3.3 ± 0.7	<0.0001
AG ratio (mean ± SD)	1.2 ± 0.4	1.3 ± 0.4	0.30	1.2 ± 0.4	1.3 ± 0.4	0.11
HBV DNA [Units/volume] (Mean ± SD)	184293.1 ± 1013807.2	207773.2 ±1130886.2	0.02	113335.1 ± 947033	266770.6 ± 1159805.8	0.15

BMI, body mass index; NAFLD, non-alcoholic fatty liver disease; NSAIDs, non-steroidal anti-inflammatory drugs; AST, aspartate aminotransferase (U/L); ALT, alanine aminotransferase (U/L); total protein (g/L); r-GT, gamma-glutamyl transpeptidase (U/L); A/G ratio, albumin to globulin ratio; HBV DNA, hepatitis virus B Deoxyribonucleic acid (IU/mL); SD, standard deviation
